# The use of clinical study reports to enhance the quality of systematic reviews: a survey of systematic review authors

**DOI:** 10.1186/s13643-018-0766-x

**Published:** 2018-08-08

**Authors:** Alex Hodkinson, Kristina Charlotte Dietz, Carol Lefebvre, Su Golder, Mark Jones, Peter Doshi, Carl Heneghan, Tom Jefferson, Isabelle Boutron, Lesley Stewart

**Affiliations:** 1 LCentre for Primary Care, Division of Population Health, Health Services Research & Primary Care, Williamson Building, Oxford Road, Manchester, M13 9PL UK; 2 Centre for Reviews and Dissemination, A/B Block, Alcuin College, University of York, York, YO10 5DD UK; 3 Lefebvre Associates Ltd, Oxford, UK; 4 Department of Health Sciences, University of York, York, UK; 5 School of Public Health, University of Queensland, Brisbane, Australia; 6 Department of Pharmaceutical Health Services Research, University of Maryland School of Pharmacy, Baltimore, MD USA; 7 Centre for Evidence Based Medicine, Nuffield Department of Primary Care Health Sciences, Primary Sciences Division, University of Oxford, Oxford, UK; 8 METHODs Team, Centre of Research in Epidemiology and Statistics Sorbonne Paris Cité, INSERM UMR, University Paris Descartes, Paris, France

## Abstract

**Background:**

Clinical study reports (CSRs) are produced for marketing authorisation applications. They often contain considerably more information about, and data from, clinical trials than corresponding journal publications. Use of data from CSRs might help circumvent reporting bias, but many researchers appear to be unaware of their existence or potential value. Our survey aimed to gain insight into the level of familiarity, understanding and use of CSRs, and to raise awareness of their potential within the systematic review community. We also aimed to explore the potential barriers faced when obtaining and using CSRs in systematic reviews.

**Methods:**

Online survey of systematic reviewers who (i) had requested or used CSRs, (ii) had considered but not used CSRs and (iii) had not considered using CSRs was conducted. Cochrane reviewers were contacted twice via the Cochrane monthly digest. Non-Cochrane reviewers were reached via journal and other website postings.

**Results:**

One hundred sixty respondents answered an open invitation and completed the questionnaire; 20/160 (13%) had previously requested or used CSRs and other regulatory documents, 7/160 (4%) had considered but not used CSRs and 133/160 (83%) had never considered this data source. Survey respondents mainly sought data from the European Medicines Agency (EMA) and/or the Food and Drug Administration (FDA). Motivation for using CSRs stemmed mainly from concerns about reporting bias 11/20 (55%), specifically outcome reporting bias 11/20 (55%) and publication bias 5/20 (25%). The barriers to using CSRs noted by all types of respondents included current limited access to these documents (43 respondents), the time and resources needed to obtain and include these data in evidence syntheses (*n* = 25) and lack of guidance about how to use these sources in systematic reviews (*n* = 26).

**Conclusions:**

Most respondents (irrespective of whether they had previously used them) agreed that access to CSRs is important, and suggest that further guidance on how to use and include these data would help to promote their use in future systematic reviews. Most respondents who received CSRs considered them to be valuable in their systematic review and/or meta-analysis.

**Electronic supplementary material:**

The online version of this article (10.1186/s13643-018-0766-x) contains supplementary material, which is available to authorized users.

## Background

The findings of clinical trials as reported in journal articles can sometimes be incomplete and misleading. There is evidence that analyses and outcomes, including both efficacy and harms, may be reported selectively such that the true effects of treatments remain hidden [1–5]. Consequently, those performing systematic reviews and meta-analyses may need to take additional steps to locate, appraise and synthesise missing or inadequately reported data in order to minimise the impact of such reporting bias.

Clinical study reports (CSRs) are produced by pharmaceutical companies during a marketing authorisation application for investigational medicinal products in the EU, Japan and the USA. They are usually written in accordance with the ‘international conference on harmonisation of technical requirements for registration of pharmaceuticals for human use’ (ICH) guideline on the structure and content of clinical study reports (ICH E3) [6]. The purpose of the ICH guidance is to assist sponsors in developing a comprehensive trial report that is complete, well-structured and easy for regulators to review when making licencing decisions [7].

CSRs often include greater detail about trial design, conduct and analysis; more complete results; and a more reliable picture of strengths and weaknesses than journal articles. Extracting data from CSRs and using these in systematic reviews and meta-analyses may therefore provide more complete information and generate more reliable effect estimates [5, 8] than using data presented in journal articles and help circumvent reporting bias [9, 10], particularly in relation to adverse events [11–13].

CSRs are becoming increasingly available and accessible following liberalisation of the European Medicines Agency (EMA) policy related to public access to documents it holds relating to market access applications [14]. Requests to the EMA for access to CSRs [15, 16] have increased from 20 requests per month during the first 2 years since 2010, to nearly 40 requests per month post the initial 2-year period. The shift towards improved transparency continued with the implementation of EMA’s Policy 0070 for publication of clinical data of medicinal products for human use [17], and other significant efforts to provide broader access to clinical trial data. These include the Yale University Open Data Access (YODA) Project [18], ClinicalStudyDataRequest.com (CSDR) [19], the Duke Clinical Research Institute (DCRI) [20], the AllTrials campaign [21] and OpenTrials.net [22]. Some of the world’s largest pharmaceutical companies including Bristol-Myers Squibb, GlaxoSmithKline, Johnson & Johnson, Lilly and Roche have committed to data sharing [23]. CSRs are now being promoted as a valuable resource in systematic reviews [5, 8, 9, 12], but because data sharing directly via companies and other various platforms is still relatively new, their existence may still be unknown to many systematic review authors, and their utility is still largely unexplored [24].

The US Agency for Healthcare Research and Quality (AHRQ) has, amongst others, noted the value of searching for ‘regulatory documents’ as a means of addressing reporting bias [25]. The term ‘regulatory documents’ can be used to describe a number of sources of information other than CSRs, including Food and Drug Administration (FDA) approval documents on the Drugs@FDA website (e.g. medical and statistical reviewer reports), European Public Assessment Reports and any document produced by, or held by, a regulatory agency.

The current version of the Cochrane Handbook, last updated in 2011, encourages authors to search for unpublished data from pharmaceutical companies, trial registers and trial result registries [26]. It does not, however, discuss searching for or considering the use of CSRs and other regulatory documents as sources of data, which could partly explain why there have so far been few Cochrane Reviews that have sought data from these sources [11, 27]. This will be addressed in the major revision currently in preparation—and is due for publication in late 2018.

Recognising the need to consider the potential value of CSRs and other regulatory documents as a potential source of data for Cochrane Reviews of pharmacological interventions, Cochrane funded a project (of which this survey was part) to explore the rationale for such use, and the readiness of Cochrane reviewers to engage with regulatory material. To assess readiness and raise awareness, we carried out a preliminary survey to gain insight to the level of understanding, familiarity with and views on the importance of CSRs and other regulatory documents. We also explored what has previously motivated authors to seek data from these sources, and barriers to using them in Cochrane and other systematic reviews. We then carried out a follow-up survey of respondents who had considered or used regulatory data in their systematic review, to explore under what circumstances they thought it most important to seek CSRs as a data source.

## Methods

We conducted two online surveys involving authors of Cochrane and other systematic reviews using the data capture tool Qualtrics [28]. The initial survey (Additional file 1) was open between 10 June and 19 September 2016. This was split into two releases, one intended for Cochrane authors and the other for authors of systematic reviews conducted outside of Cochrane. A second (follow-up) survey (Additional file 2) was open between 21 April and 31 May 2017.

The survey design included closed and open-ended questions. Response options were discussed, drafted and refined by the team. Question types included multiple choice (check one/check all), free-text and comments. ‘Other (specify)’ responses were offered to capture more detailed information that could not be collected using structured multiple choice questions. Pilot testing of the survey logic and flow was performed by AH and checked by members of the research team and four independent researchers. Revisions were made where necessary.

Ethics approval for the survey was granted by the University of York Health Sciences Research Ethics and Integrity Governance Committee.

### Sample selection

The release of the initial survey intended for Cochrane review authors was first announced in the Cochrane Digest, which was emailed to all Cochrane authors on 10 June 2016. It was then mentioned again in the Cochrane Digest 2 weeks later. The release intended for authors of non-Cochrane reviews was first advertised on the University of York Centre for Reviews and Dissemination web site on the 25 June 2016 and then on the *Systematic Reviews* journal web site. Links to this were also shared via social media. The follow-up survey was sent only to those Cochrane respondents who had previously considered or used regulatory data in their systematic review and who had agreed during the first survey to participate in the follow-up. Although several authors of this manuscript have previously used data from regulatory documents, purposely none participated in either survey.

### Domains of interest

The first survey questionnaires (Additional file 1) were accessed via three separate links within the adverts corresponding to the respondent’s experience and understanding of the regulatory process aiming to capture those who had:

1. ‘Requested’ (i.e. had used data from CSRs in their review, or had received data but decided not to include it, or were still awaiting for data)
2. ‘Considered’ using but not requested access to CSRs
3. ‘Never considered’ the use of regulatory documentation such as CSRs

Respondents who had previously ‘requested’ CSRs or other regulatory documents were asked to explain their reasons for seeking and for using (or not using) the data, the source and type of documents obtained and how the data were used in the review. They were also asked to describe any difficulties in using provided documents and data, along with their views on the overall importance of seeking data from these sources. Respondents that had only ‘considered’ seeking regulatory documents were asked about what sources they had considered utilising and why they had decided against it; and whether they thought this decision could have impacted on the outcome of their review. Other domains of interest captured were the respondents’ views on the barriers to using data from regulatory documents and what could be done to promote and support the use of such data in future systematic reviews. Those who had never considered the use of regulatory documentation were asked for their views on their potential value and whether they might be encouraged to consider these data in the future.

The follow-up survey (Additional file 2) explored what factors might be considered most influential when deciding whether to look beyond the information presented in journal articles and to seek data from CSRs or other regulatory documents for use in a systematic review. We drew up an initial list of criteria on which respondents were asked to comment (Table 1). Likert scales (very important, important, less important, not important and unsure) were used to grade the importance of each criterion. Respondents were also asked to identify any additional criteria that would be important when deciding whether to seek data from regulatory documents.

**Table 1:** Important criteria when considering data from clinical study reports and/or other regulatory data

Criteria	Description of criteria
1	Monetary cost of the intervention on the healthcare budget (i.e. considering both the price of a course and the number of people in the population that are being—or will be—treated)
2	Burden of disease of the indication this product is meant to treat/prevent
3	How many people are using or likely to use this product?
4	Product new to the market?
5	Product from a new drug class or has a new mechanism of action
6	Has important interactions with other drugs (e.g. drug-drug interactions)
7	High proportion of RCTs evaluating this product are industry funded
8	Prominent claims of safety and/or efficacy advantage of this product over currently available treatments
9	High degree of media attention surrounding this product
10	High proportion of trials of this product are unpublished
11	Post-marketing surveillance has identified safety concerns?
12	Important or standard outcome measures (also known as ‘endpoints’) have not been published
13	Concerns regarding a lack of published data on potential harms of the product
14	Marketing authorisation based on surrogate outcomes (rather than clinical outcomes)
15	When protocol(s) are publicly available
16	When statistical analysis plan(s) publicly available
17	Known errors or concerns about trial publications of this product
18	Important discrepancies between the journal publication and the trial registry entry?

### Data analysis

Descriptive statistics were used to express quantitative responses including number(s), frequencies and percentages. Verbatim responses were discussed within the team and then tabulated. Responses were generally short and wide-ranging and coded into categories by two research team members (AH, KCD).

**Fig. 1:**
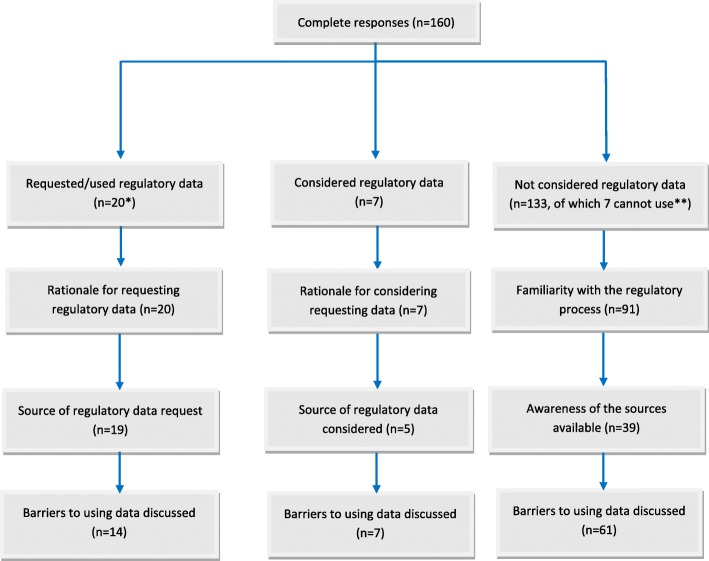
Flow diagram of combined survey responses with responses in each domain of interest

Since the two first survey questionnaires intended for (a) Cochrane and (b) other systematic review authors were the same, and because some respondents answered the Cochrane questionnaire based on non-Cochrane reviews and vice versa, and as there were few responses to the non-Cochrane version, we combined and analysed responses to both together (Fig. 1). We firstly checked that there was no duplication or double counting of reviews. We obtained publications for the systematic reviews to which respondents referred (if provided by the respondents in the survey). This enabled us to confirm whether reviews were Cochrane reviews or not, and to help resolve ambiguity in free-text responses (e.g. to confirm which sources provided data and determine how the data were used in a review). Authors were contacted directly by email to confirm any other questions we had about their review.

## Results

A total of 160 respondents completed the first (Cochrane and non-Cochrane) surveys (Fig. 1). Most respondents (93%) were either a Cochrane review author or an editor, 70% worked in academia, 40% were clinicians and 15% were involved in methods research. Of the 160 respondents, 20 (13%) had previously requested or used regulatory data in their review (13 in a Cochrane review and 7 in a non-Cochrane review), 7 (4%) had considered but not used regulatory data and 133 (83%) had never considered using regulatory data.

In the follow-up survey, all 20 respondents who had requested or used regulatory data in a systematic review explained the rationale for making the request, 19 (95%) provided information on where data were requested from and 14 (70%) expressed an opinion about the type of barriers involved. All 7 respondents who considered using regulatory data but did not go on to seek it explained the rationale for doing so, and 5 (71%) gave the primary source of the data under consideration and all responded about potential barriers. For the 133 respondents that had never considered using regulatory data, 91 (68%) indicated that they were familiar with the regulatory process and types of documents produced, 39 (29%) were aware of where they might be able to access regulatory documents such as CSRs and 61 (46%) believed that there are barriers to accessing and using data from these sources.

### Rationale for seeking data

For the 20 respondents who had requested or used regulatory data, 15 (75%) believed that regulatory data should be used in systematic reviews and 5 (25%) said that they should be used in some cases (Additional file 3: Table S1 ). Seeking regulatory data was mainly driven by concerns about reporting bias (specifically outcome reporting bias, (*n* = 11), publication bias (*n* = 5) and potential for missing data (*n* = 2) or underreporting of harms (*n* = 3). The same concerns were raised by respondents who had considered but not sought regulatory data, with 3/7 mentioning outcome reporting bias, and 2/7 underreporting of harms.

For respondents who had never considered using regulatory data, 66/133 (50%) agreed that they should be used in some cases, 43 (32%) said they should definitely be used, 17 (13%) said they should not be used and 7 (5%) said they were unsure about using regulatory data but did not provide any reasons. The reasons given by the 17 respondents who said that regulatory data should not be used were (*n* = 9) because the interventions explored in their reviews were non-pharmacological, (*n* = 5) because of lack of guidance on how to find and use these data and (*n* = 3) because of the time required to obtain the data.

Eight out of 133 (6%) respondents who had never considered using regulatory documents indicated that they had a detailed understanding and 83 (62%) a basic understanding of the regulatory process (Additional file 3: Table S2). However, 42/133 (32%) respondents said that they had no understanding of the regulatory process or the documentation involved. The majority said that they were aware of the ongoing debates and initiatives for improved access to clinical trial data, specifically referring to the AllTrials initiative [21], the Cochrane review of neuraminidase inhibitors (which was based entirely on regulatory data) [9] and other publications such as Ben Goldacre’s *Bad Science* and *Bad Pharma*. One respondent said that they had been involved in crafting the EMA-led public deliberations regarding the Policy 0070 in 2014, for access to clinical trial data [17].

### Source of evidence

**Fig. 2:**
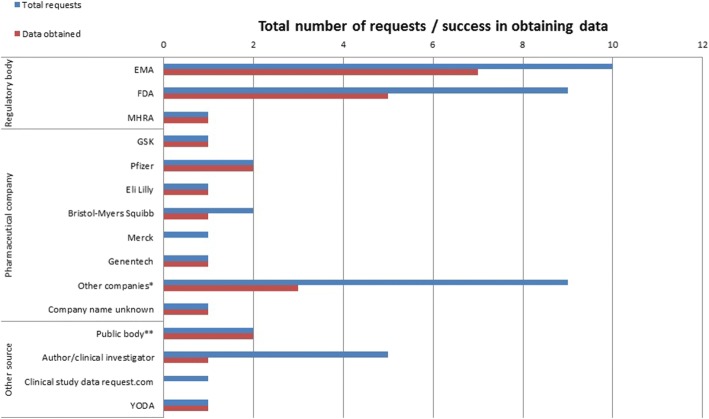
Sources of data for the respondents who requested regulatory/non-regulatory data and the success rate obtaining the data. *Larger companies include GSK (*n* = 1 request (1: successful request)), Pfizer (*n* = 2 (2)), Eli Lilly (*n* = 1 (1)), Bristol-Myers Squibb (*n* = 2 (1)), Merck (*n* = 1 (0)), Genentech (*n* = 1 (1)). **Smaller companies include (2) Helsinn, (2) Schering-Plough, (1) Salix Pharmaceuticals, (1) PharmaSwiss, (1) Cubist Pharmaceuticals, (1) Pharmaxis, (1) Santhera. **(1) Request was made to the US’s National Institute for Occupational Safety and Health (NIOSH) and the other to Health Canada

Figure 2 shows where data access requests were made (including the respondents who made multiple requests to different sources). In total, there were 47 requests, of which 19 (40%) were made to regulatory agencies of which 10/19 (53%) were to the EMA with seven of the requests successful in obtaining data; and 9/19 (47%) were to the US Food and Drug Administration (FDA) where five requests were successful. Eighteen out of 47 (38%) requests were made directly to pharmaceutical companies; 8 to larger companies where six (75%) requests were successful, and nine to smaller companies where 3 (33%) requests were successful (Fig. 2). One respondent noted a request made to the National Institute for Occupational Safety and Health in the USA [29] for summary adverse events data, and another to Health Canada. Only two requests were made to the data sharing websites, Clinical Study Data Request (CSDR) and YODA, where each was successful in obtaining the data.

Amongst the 20 respondents who requested regulatory data (Table 2), 16 had obtained and used the data in their review, two had not yet received the data and their review was ongoing at the time of completing the survey. One respondent said they had received only baseline data and therefore did not include it in their review, and one reported being unable to access the full data because the study was stopped early due to reports of unexpected side-effects. Clinical study reports were acquired by 12 (60%) of the respondents. Five obtained Medical and Statistical Reviews from the FDA, two obtained European Public Assessment Reports (EPARs), and one used other regulatory material including a protocol, case report forms and adverse reaction reports. Of the respondents who obtained CSRs, nine (9/12 (75%)) had used data from them to enable inclusion of unpublished trials in their meta-analyses (*n* = 2) and to supplement published data (*n* = 7). The other two respondents who obtained CSRs used them in a narrative synthesis; one within a NICE Single Technology Appraisal (STA).

**Table 2:** Description of data obtained and how they were used in the systematic reviews

Survey reply	Source of data request(s)	Data obtained	Type of regulatory data/document(s) obtained	Included in meta-analysis	Description of how data were used
1	Author, manufacturer	Yes	CSRs	Yes	‘Summary statistics provided or extracted from the extra documentation were incorporated into meta-analysis’
2	Unknown	Yes	CSRs	Yes	‘Quantitative data about side effects were included’
3	EMA, FDA	Yes	CSRs	No	‘Data were not used in meta-analyses, but rather in a narrative form instead’
4	EMA, FDA	Yes	EPARs and Medical Reviews	No	‘Data was used to describe the number of studies and the number of studied drugs in results of search criteria’
5	EMA, FDA, Multiple drug companies	Yes	FDA and EMA reports, Poster	Yes	‘To add data on studies not aware of, and to add outcomes to a published study that were not itemised in the journal publication’
6	Clinical investigator, EMA, sponsor	No^β^	No data were obtained	N/A	‘Not provided by pharmaceutical sponsor, possibly because study stopped early due to unexpected side effects, and raw data may never have been compiled.’
7	FDA, Health Canada, NIOSH	Yes	Adverse event reports	No	‘The data did not provide some of the detail we would have liked, such as indication for the drug, dosing etc. We summarized the results in narrative form but did not include in the quantitative analyses of the data we retrieved from published studies’
8	Clinical investigator, medical director of company	Yes	CSRs	Yes	‘Assessed quality of the studies and extracted data for use in forest plots and description’
9	Clinical Study Data Request, EMA, FDA	Other*	Case report forms	N/A	‘N/A as data not received’
10	Clinical investigator, EMA, Pharmaceutical company	Other^¥^	Details of trial participants at start of trial (baseline data and info about randomisation)	No	‘Only data at start of trial was available’
11	EMA, GSK and FDA	Yes	Clinical and Statistical reviews at FDA, CSRs	Yes	‘We checked the data for consistency (across multiple published and unpublished sources) and reported in the systematic review the most accurate and conservative estimates. If needed, we contacted authors for confirmation’
12	Pharmaceutical company	Yes	CSRs, IPD	Yes	‘Data from CSRs & IPD were used in evidence synthesis’ ‘We know patient level data exists but we were not given access to it despite trying’
13	Pfizer	Other*	CSRs	N/A^€^	‘Extraction of data from Pfizer Medical Information Report’
14	EMA, FDA	Yes	CSRs, protocol with appendices	Yes	‘We extracted, compared and used the aggregated effect estimates data for predefined outcomes’
15	Helsinn, Merck and Pfizer	Yes	CSRs	Yes	‘Where possible incorporated it as more likely to be the correct data than what was published’
16	EMA, FDA	Yes	FDA medical and statistical reviews	Yes	‘Performed data extraction from these sources. Compared with data from published sources’
17	EMA, NIOSH	Yes	N/A	No	‘Excluded studies’
18	FDA	Yes	CSRs, FDA reports and IPD	Yes	‘Data was used in place of publication’
19	YODA	Yes	CSRs	Yes	‘Data were used in network meta-analyses’
20	Bristol-Myers Squibb, Genentech, Schering-Plough	Yes	CSRs	No	‘In narrative synthesis. However, some of the data/text needed to be removed before the final technology assessment report is published under the confidentiality agreement’.

*N/A* not applicable, *FDA* Food and Drug Administration, *EMA* European Medicine Agency, *NIOSH* The National Institute for Occupational Safety and Health

^β^Response: ‘data not provided by pharmaceutical sponsor possibly because study was stopped early due to unexpected side effects and therefore the raw data may not have been complied’

*Still awaiting data/updating review

^¥^Intended data requested was not available

^€^Intend to incorporate data in a meta-analysis

Of the 133 respondents who had never considered accessing regulatory data, 117 (88%) said they were not aware (or were unsure) of where to access such material (Additional file 3: Table S3). Sixteen (12%) respondents said that they were aware of at least one possible source of regulatory data. The EMA and FDA were the two sources most commonly noted, but other regulatory agencies mentioned were The Health Products Regulatory Authority of Ireland, Pharmaceuticals and Medical Devices Agency of Japan and Therapeutic Good Administration Department of Health Australia. Other sources mentioned but not considered to be specific to regulatory data included the trial registries (ClinicalTrials.gov and the ISRCTN register). The clinical study data request sharing platform was considered by only one respondent. Pharmaceutical companies and ethics committees were also mentioned as other sources of data.

### Barriers

Survey respondents were asked to express views on the real or perceived barriers to accessing and using regulatory data including CSRs (Table 3). Over 70% of the authors, who had used, requested or at least considered regulatory data, reported there to be barriers compared to 50% for respondents that had not considered the use of such data. Specifically, for those who had requested data, 14/20 (70%) identified barriers including ‘restricted and limited sharing of trials data’, and the ‘time-constraints involved [in] searching and requesting the data’, ‘the lack of experience on extracting data and [lack of] statistical guidance when including in a review’, ‘how and where to search for individual trials’ and one mentioned ‘concerns over the quality of the data compared to the journal publication’. For respondents who had only considered (but not requested) regulatory data, 6/7 (86%) indicated similar barriers. For respondents who had not considered using regulatory data, 67/133 (50%) believed there to be barriers, whilst 56/133 (42%) were unsure. The barriers noted by this group were also similar in citing ‘cost’, ‘time and resources required in searching and requesting for data’ and also ‘limited access for the peer reviewer’ which we understood to mean that data included in regulatory documents were unpublished and had therefore not been peer reviewed.

**Table 3:** Barriers when seeking regulatory data for use in a Cochrane review

	Requested/used regulatory data	Considered regulatory data	Not considered regulatory data
Survey question	Total no. of responses: n (% of total responses)		
Are there any barriers to using regulatory data?	*n* = 20	*n* = 7	*n* = 133
Yes	14 (70)	6 (86)	67 (50)
No	2 (10)	0 (0)	10 (8)
Unsure	4 (20)	1 (14)	56 (42)
What were these barriers?	*n* = 14	*n* = 5	*n* = 60
Restricted and limited sharing of data	8	4	31
Time-constraints	6	2	17
Lack of experience (inc. statistical)	4	1	21
Identifying/searching for trials	2	1	13
Quality of data	1	0	12
Cost	0	1	1
Effort/resources required	0	0	5
Limited access for peer reviewers*	0	0	1

*This referred to peer reviewers not having access to regulatory data during the peer review stage

### Criteria considered important for using regulatory data

**Fig. 3:**
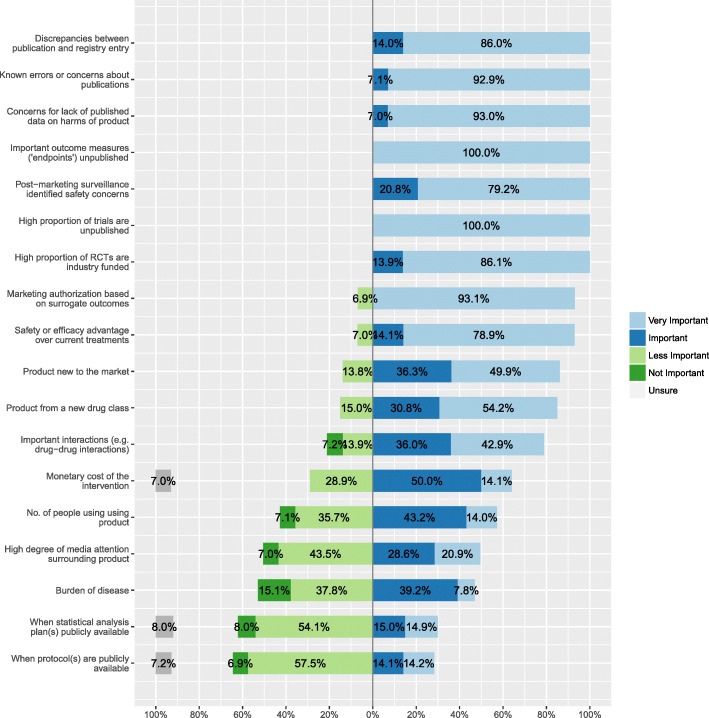
Criteria considered most important when considering using regulatory data (*n* = 14)

Results of the follow-up, targeted survey designed to identify the main reasons or triggers for authors seeking and using data from regulatory documents, are shown in Fig. 3. This was sent to the 21 first survey respondents who had agreed to participate in a follow-up (6 respondents to the first survey were unwilling to participate in a follow-up). Fourteen of the 21 (66%) provided a response. The following criteria were considered of most importance (i.e. ‘very important’ or ‘important’) by all respondents in deciding when it is most important to use regulatory data in a systematic review: ‘discrepancies between publication and registry entry’, ‘known errors or concerns about publications’, ‘concerns for a lack of published data on harms of product’, ‘important outcome measures (‘endpoints’) unpublished’, ‘safety concerns identified in post-marketing surveillance’, ‘high proportion of trials unpublished and/or industry funded’.

Between 9 (64%) and 13 (93%) respondents considered ‘marketing authorisation based on surrogate outcomes’, ‘safety or efficacy advantage over current treatments’, ‘product new to the market or from a new drug class’, ‘important drug-drug interactions’ and ‘monetary cost of the intervention’ to be important.

The two criteria deemed less important (by 6 (43%) to 9 (64%) of authors) included the public availability of ‘statistical analysis plans’ and of ‘protocols’.

Respondents expressed divided opinion about whether ‘number of people using the product’, ‘high degree of media attention surrounding the drug’ and ‘burden of disease’ were important ‘triggers’.

Respondents suggested as other ‘triggers’ for seeking and using of regulatory data: ‘the lack of clarity on published trials’ and ‘when a small number of trials are available’. It was also noted that ‘regulatory data were irrelevant for non-pharmacological intervention reviews (e.g. surgical techniques, psychological interventions and psychical therapy) and were therefore unable to use CSRs.’

## Discussion

### Summary of findings

In this survey, only 27/160 (17%) systematic review authors had used, requested or considered using regulatory data in their review and 133/160 (83%) had never considered using such data. Respondents who had requested regulatory documents had mainly sought these from the EMA and the FDA. Other requests were made to individual pharmaceutical companies, but only one was made to a data sharing platform. Respondents also described seeking data from the clinical investigators or authors of published trials in their responses, although these are clearly not usual sources of regulatory documents, which may indicate unfamiliarity with and a misunderstanding of the question posed as being about any ‘unpublished data’ rather than specifically being focused on regulatory documents.

Clinical study reports were acquired by 12/20 (60%) of the respondents requesting data, but other regulatory documents including Medical and Statistical Reviews from the FDA (5/20 (25%)), European Public Assessment Reports (EPARs) (2/20 (10%)), and protocols, case report forms and post-marketing adverse reaction reports were also obtained. For the respondents who obtained CSRs, 9/12 (75%) had used the data in their review, in order to include unpublished trials in their meta-analyses and to supplement published data. Two of the respondents were still waiting for the data, one respondent noted that the pharmaceutical company could not provide the data because the study for which the request was made was stopped early due to reports of unexpected side-effects and another respondent reported that only baseline data were provided.

At least two thirds of the respondents who requested or considered utilising regulatory data reported a number of barriers to inclusion of such data in Cochrane reviews including restrictions on accessing trial data, the excessive time involved when waiting for data to be released and the resources, costs and effort required when incorporating the data in a review.

The criteria considered to be most important in triggering decisions to seek regulatory data include situations where there are discrepancies between a study publication and its corresponding trial registry entry, known errors or concerns about publications including a lack of data on harms and where important outcome measures (‘endpoints’) are not reported. Safety concerns identified in post-marketing surveillance, situations where a high proportion of trials are unpublished and/or industry funded, and cases where marketing authorization was based on surrogate outcomes were also considered important indicators of when it would be valuable to seek regulatory data. The lack of availability of the trial protocol and statistical analysis plan and media attention about the drug were considered to be ‘less important’. The cost of the intervention, disease burden, population size and characteristics of the intervention (new to market, interactions with other drugs) were of more mixed opinion of importance amongst authors.

### Comparison with other research

A previous study [11] exploring the experiences of Cochrane review authors when searching for, gaining access to and using unpublished data found that a large proportion of Cochrane review authors had searched for unpublished data. Over half (913/1656 (55.1%)) of those who searched for unpublished data were successful in finding it, and over 81% (651/794) who sought these data went on to use them in their review. In that study, most of the unpublished data were obtained from ‘trialists or investigators’. Of 794 author requests in their study, 403/794 (51%) sought summary data (e.g. mean, standard deviation, sample size), 226/794 (29%) missing outcomes (e.g. quality of life), 163/794 (21%) individual participant data (IPD), 96/794 (12%) results of alternative analysis (e.g. intention to treat), 67/794 (8%) data on harms and 45/794 (6%) CSRs. Data from manufacturers were less frequently used in these reviews. One of the concerns outlined by the authors was that searching for unpublished data was time consuming, which aligns with the opinions expressed by respondents in our survey. Despite the perceived importance of CSRs in providing information about adverse effects, a recent study found that of 348 systematic reviews on adverse effects published in 2014, not one of the reviews had stated that they searched for or included CSRs [27].

Another study [30] provided in-depth descriptions of some of the experiences of researchers carrying out systematic reviews when searching for and gaining access to unpublished data. That work aimed to provide guidance on best practices for identifying, obtaining and using unpublished data from a variety of sources, but did not consider regulatory documents. The results suggested that authors differed in their understanding of what was meant by *unpublished data*, including specific outcomes and methodological details. They also reported that data requests were often seen as time consuming and that including such data was considered to be labour-intensive. There was agreement, however, by the majority of authors that searching for and considering unpublished data in systematic reviews was important for helping to improve the general public health.

### Accessing regulatory data

Based on interactions with the community, it seems that many researchers undertaking systematic reviews are still unaware of the various data sharing platforms that provide access to regulatory trial documents and datasets. This may be partly because such data sharing platforms are relatively new and evolving. Furthermore, the limited formal guidance available to systematic review authors explaining how to identify and access regulatory data might also explain why they are rarely used or considered. For example, as noted above, the current version of the Cochrane Handbook does not currently discuss regulatory documents and the data that these might contain, where to find these data, or how to include them [31]. In our study, participants were asked what could be done to promote and support greater use of CSRs and other regulatory documents. Most agreed that there is a need for greater understanding about these documents and for guidance on how to search for and access such data. Some mentioned the need for statistical guidance on how to include the data in evidence synthesis, even though the type of (aggregate) data that these documents contain are largely no different to the type of data presented in journal articles and do not need to be handled and analysed differently (data from patient narratives or case report forms included within a CSR will need different handling). There were also concerns about how to interpret highly statistical content within the documents, e.g. efficacy and safety listings data which may require statistical/software expertise to help extract and organise the data.

### Limitations of study

As most of the survey questions captured free-text response(s), the replies were varied and some were unclear or lacked enough detail to understand fully. However, responses were discussed by two team members (AH and KCD) who agreed upon an appropriate classification of response.

Although this survey concerned regulatory documents, and in particular CSRs, it was apparent even from the relatively low numbers who responded that some may have misunderstood some of the questions posed. The term ‘regulatory data’ was not defined in the survey, as we were interested in learning how respondents interpreted this and this may explain why some authors who had used other types of data (e.g. IPD or other summary data *obtained from trial authors*) responded in the survey. In a number of the responses, authors had made multiple data requests for regulatory data and other data, but it was not always clear in their responses which data or document sources were actually used in their systematic review or meta-analysis. The review references provided, or obtained by contacting the authors, helped to resolve some of these uncertainties about how the data were used in reviews.

The initial survey of Cochrane authors was advertised twice in the Cochrane methods digest, which is circulated by email to the whole Cochrane community. We do not know how many people actually received and opened the email, and consequently how many people read the invitation to opt into the survey. This might explain the low response rate (2.2% calculated using the known figure of 7273 registered Cochrane reviews over the last 2 years as the denominator) compared with that achieved in the survey by Schroll et al. [11] (37%) which sent invitations to participate directly to review authors. The Schroll survey may also have achieved higher numbers of responses because most Cochrane authors understand the concept of using ‘unpublished data’ whereas fewer may have been familiar with the idea of using regulatory documentation. Furthermore, the use of unpublished data is relevant to all systematic reviews, whilst regulatory documents apply only to reviews of interventions that have made, or intended to make, an application for market authorisation. As respondents in our survey may have been more likely to have a greater understanding of the regulatory process and documents produced, than authors who did not participate in our survey, our sample might not be representative of all authors of systematic reviews. The survey cannot be used, and indeed was not intended, to draw any conclusions about the proportion of reviewers accessing CSRs. Rather it aimed to gain insight into the level of familiarity with regulatory sources of data, particularly amongst Cochrane authors, and to get some indication of the potential level of ‘buy in’ to future encouragement to use these sources in Cochrane Reviews, and what support may be needed to facilitate this.

## Conclusions

The results from this survey show that data from CSRs and other regulatory documents are being used in a small number of Cochrane Reviews. The survey revealed that the vast majority of respondents thought that accessing and using CSRs and other regulatory documents in systematic reviews was important, suggesting that the Cochrane community may be ready and willing to engage with this source of evidence. The time taken and resource needed to request, receive and use the data was cited as a major barrier, as was the lack of formal guidance on access to and use of documents produced for regulatory purposes. There is a pressing need to develop materials to help review authors identify questions and topics where using regulatory data is likely to matter most and which should therefore adopt and invest in such an approach, and to help them navigate regulatory documents and incorporate data from them in Cochrane and other systematic reviews.

## Additional files

Additional file 1: Three survey links for initial survey. (DOCX 22 kb)

Additional file 2: Follow-up survey. (DOCX 16 kb)

Additional file 3: **Table S1**. Characteristics of respondents and their experiences with regulatory data. **Table S2**.Characteristics of respondents who have never considered using regulatory data. **Table S3**. Possible sources for respondents who had not considered regulatory data. (DOCX 17 kb)

## References

[1] McGauran N, Wieseler B, Kreis J, Schüler Y-B, Kölsch H, Kaiser T (2010). Reporting bias in medical research—a narrative review. Trials.

[2] Page MJ, McKenzie JE, Kirkham J, Dwan K, Kramer S, Green S, Forbes A (2014). Bias due to selective inclusion and reporting of outcomes and analyses in systematic reviews of randomised trials of healthcare interventions. Cochrane Database Syst Rev.

[3] Dwan K, Altman DG, Arnaiz JA, Bloom J, Chan AW, Cronin E, Decullier E, Easterbrook PJ, Von Elm E, Gamble C (2008). Systematic review of the empirical evidence of study publication bias and outcome reporting bias. PLoS One.

[4] Dwan K, Gamble C, Williamson PR, Kirkham JJ, for the Reporting Bias Group (2013). Systematic review of the empirical evidence of study publication bias and outcome reporting bias—an updated review. PLoS One.

[5] Eyding D, Lelgemann M, Grouven U, Harter M, Kromp M, Kaiser T, Kerekes MF, Gerken M, Wieseler B (2010). Reboxetine for acute treatment of major depression: systematic review and meta-analysis of published and unpublished placebo and selective serotonin reuptake inhibitor controlled trials. BMJ.

[6] International Conference on Harmonisation of Technical requirements for registration of Pharmaceuticals for Human use (ICH): Structure and Content of Clincial Study Reports E3. [http://www.ich.org/fileadmin/Public_Web_Site/ICH_Products/Guidelines/Efficacy/E3/E3_Guideline.pdf]. 10.1111/j.1365-2125.1994.tb05705.x PMC1364893 8054244

[7] Doshi P, Jefferson T (2013). Clinical study reports of randomised controlled trials: an exploratory review of previously confidential industry reports. BMJ Open.

[8] Le Noury J, Nardo JM, Healy D, Jureidini J, Raven M, Tufanaru C, Abi-Jaoude E (2015). Restoring study 329: efficacy and harms of paroxetine and imipramine in treatment of major depression in adolescence. BMJ.

[9] Jefferson T, Jones M, Doshi P, Del Mar C (2009). Neuraminidase inhibitors for preventing and treating influenza in healthy adults: systematic review and meta-analysis. BMJ.

[10] Wieseler B, Wolfram N, McGauran N, Kerekes MF, Vervölgyi V, Kohlepp P, Kamphuis M, Grouven U (2013). Completeness of reporting of patient-relevant clinical trial outcomes: comparison of unpublished clinical study reports with publicly available data. PLoS Med.

[11] Schroll JB, Bero L, Gotzsche PC (2013). Searching for unpublished data for Cochrane reviews: cross sectional study. BMJ.

[12] Hodkinson A, Gamble C, Smith CT (2016). Reporting of harms outcomes: a comparison of journal publications with unpublished clinical study reports of orlistat trials. Trials.

[13] Golder S, Loke YK, Wright K, Norman G (2016). Reporting of adverse events in published and unpublished studies of health care interventions: a systematic review. PLoS Med.

[14] Gotzsche PC, Jorgensen AW (2011). Opening up data at the European Medicines Agency. BMJ.

[15] Doshi P, Jefferson T (2016). Open data 5 years on: a case series of 12 freedom of information requests for regulatory data to the European Medicines Agency. Trials.

[16] Wieseler B, McGauran N, Kerekes MF, Kaiser T (2012). Access to regulatory data from the European Medicines Agency: the times they are a-changing. Syst Rev.

[17] European Medicines Agency (EMA) Policy on publication of clinical data for medicinal products for human use. Policy/0070. 2 October 2014 EMA/240810/2013. [http://www.ema.europa.eu/docs/en_GB/document_library/Other/2014/10/WC500174796.pdf].

[18] The YODA Project: Forging a unified scientific community. [http://yoda.yale.edu/].

[19] ClinicalStudyDataRequest.com. [https://clinicalstudydatarequest.com/].

[20] Duke Clinical Research Institute: From thought leadership to clinical practice. [https://www.dcri.org/].

[21] +AllTrials. All Trials Registered | All Results Reported. [http://www.alltrials.net/].

[22] OpenTrials. All the data on all the trials, linked. [https://opentrials.net/].

[23] Goldacre B, Lane S, Mahtani KR, Heneghan C, Onakpoya I, Bushfield I, Smeeth L (2017). Pharmaceutical companies’ policies on access to trial data, results, and methods: audit study. BMJ.

[24] Doshi P (2014). From promises to policies: is big pharma delivering on transparency?. BMJ.

[25] Methods Guide for Effectiveness and Comparative Effectiveness Reviews. AHRQ Publication No. 10(14)-EHC063-EF. Rockville, MD: Agency for Healthcare Research and Quality. January 2014. [https://effectivehealthcare.ahrq.gov/sites/default/files/pdf/cer-methods-guide_overview.pdf]. 21433403

[26] Lefebvre C, Manheimer E, Glanville J. Chapter 6: searching for studies. In: JPT H, Green S, editors. Cochrane handbook for systematic reviews of interventions version 5.1.0 (updated March 2011): The Cochrane Collaboration; 2011. http://training.cochrane.org/handbook.

[27] Golder S, Loke YK, Wright K, Sterrantino C (2016). Most systematic reviews of adverse effects did not include unpublished data. J Clin Epidemiol.

[28] Qualtrics. Software to manage the entire customer experience—from surveys, to insights, to action. [https://www.qualtrics.com/].

[29] The National Institute for Occupational Safety and Health (NIOSH). [https://www.cdc.gov/niosh/index.htm].

[30] Wolfe N, Gøtzsche PC, Bero L (2013). Strategies for obtaining unpublished drug trial data: a qualitative interview study. Syst Rev.

[31] Higgins JPT, Green S, editors. Cochrane handbook for systematic reviews of interventions version 5.1.0 [updated March 2011]: The Cochrane Collaboration; 2011. Available from http://training.cochrane.org/handbook

