# Supplementary material for: The use of clinical study reports to enhance the quality of systematic reviews: a survey of systematic review authors
Source: Syst Rev. 2018 Aug 8;7:117. doi: 10.1186/s13643-018-0766-x (PMC6083614; doi:10.1186/s13643-018-0766-x)
Supplement: Supplementary file 1 — Three survey links for initial survey. (DOCX 22 kb) [file 13643_2018_766_MOESM1_ESM.docx]

**Additional file 1: Three survey links for initial survey**

1. **Used/Requested regulatory data**

This survey is for Cochrane authors who have previously requested regulatory information/data for use in a systematic review. If this criterion does not apply, please return to the invitation to participate and click on the appropriate link. Participation in this survey is voluntary

Q1 How many systematic reviews have you requested clinical trial data/information from regulatory sources for (we will ask you about your most recent review)?

- 1 (1)
- 2 (2)
- 3 (3)
- >3 (4)

**(A) About your review**

Q2 A1. Please provide the bibliographic detail (citation) for your most recent review.  (Full details would be appreciated but a DOI is sufficient)

Q3 A2. Please provide your main reason(s) for requesting the data/information from regulatory sources

Q4 A3. Where did you request the data/information from? (If requests were made to more than one place please list them all). Were requests successful?

|  | Source (1) | Data obtained (Y/N) (2) | Comment (3) |
| --- | --- | --- | --- |
| Request 1 (1) |  |  |  |
| Request 2 (2) |  |  |  |
| Request 3 (3) |  |  |  |

Q5 A4. Did you use the data obtained within your Cochrane review?

- Yes (1)
- No (2)
- Other (please specify) (3) ____________________

If No Is Selected, Then Skip To A5. Why were the obtained data not usedIf Yes Is Selected, Then Skip To A6. Which sources of data were used i...If Other (please specify) Is Selected, Then Skip To A6. Which sources of data were used i...

Q6 A5. Why were the obtained data not used?

If A5. Why were the obtained d... Is Displayed, Then Skip To A11. Any further comments about this ...

Q7 A6. Which sources of data were used in the review (check all that apply)?

- Clinical study reports (CSRs) (1)
- Other documents (Please specify) (2) ____________________
- Individual participant level datasets (3)
- Other(s) (Please specify) (4) ____________________

Q8 A7. How did you use the data within your review?

Q10 A8. Were there any complications when using the data in this review?

- Yes (1)
- No (2)

If Yes Is Selected, Then Skip To A9. what were they?If No Is Selected, Then Skip To A10. How important was it to use thes...

Q9 A9. What were they?

Q10 A10. How important was it to use these data in the review?

Q11 A11. Any further comments about this review?

**(B) Using regulatory data in Cochrane reviews generally**

Q12 B1. Do you think regulatory data should be used in Cochrane reviews?

- Yes (1)
- In some cases (2)
- No (3)
- Unsure (4)

If Unsure Is Selected, Then Skip To End of Block

Q13 B2. Why do you think this?

Q14 B3. Are there barriers to using regulatory data in Cochrane reviews?

- Yes (1)
- No (2)
- Unsure (3)

If Yes Is Selected, Then Skip To B4. What are these barriers and what ...If No Is Selected, Then Skip To B5. What could be done to promote and...If Unsure Is Selected, Then Skip To B5. What could be done to promote and...

Q15 B4. What are these barriers and what would help overcome them?

Q16 B5. What could be done to promote and support greater use of regulatory data in Cochrane reviews?

**(C) Other comments**

Q17 C1. Any other comments

**(D) Your background and role**

Q18 D1. Cochrane role (please check all that apply)

- Reviewer/author (1)
- Editor (2)
- Trial Search Coordinator (3)

Q19 D2. Your background (please check all that apply)

- Statistician (1)
- Methodologist (2)
- Clinician (3)
- Academic researcher (4)
- Other (please specify) (5) ____________________

**(E) Next steps**

Q20 We plan to collate information about the main triggers for requesting regulatory data and then consult on how important each of these are

Q21 E1. Would you be willing to participate in a short follow-up survey about this?

- Yes (1)
- No (2)

Q22 E2. Would you like to receive a copy of our final report?

- Yes (1)
- No (2)

Q23 If yes (to either survey or report) please provide your name and email address so that we can contact/send to you

Q24 Name

Q25 Email Address

Thank you for your participation

1. **Considered regulatory data**

This survey is for Cochrane authors who have considered using regulatory data/information in their review but decided against it. If this criterion does not apply, please return to the invitation to participate and click on the appropriate link. Participation in this survey is voluntary

Q1 for how many systematic reviews did you actively consider using clinical trial data/information from regulatory sources (we will ask you about your most recent review)?

- 1 (1)
- 2 (2)
- 3 (3)
- >3 (4)

**(A) About your review**

Q2 A1. Please provide the bibliographic details (citation) for your most recent review.  (Full details would be appreciated but a DOI is sufficient)

Q3 A2. Please provide your main reason(s) for considering requesting clinical trial data/information from regulatory sources

Q4 A3. Where did you consider requesting the data from, were specific sources considered?

- Yes (1)
- No (2)

If Yes Is Selected, Then Skip To A4. Please list all sources considered If No Is Selected, Then Skip To A5. Why did you decide not to seek/re...

Q5 A4. Please list all sources considered

|  | Name (1) | Comment (2) |
| --- | --- | --- |
| Source 1 (1) |  |  |
| Source 2 (2) |  |  |
| Source 3 (3) |  |  |

Q6 A5. Why did you decide not to seek/request the clinical trial data/information from regulatory sources?

Q7 A6. Do you think that this impacted on your review?

- Yes (1)
- No (2)
- Unsure (3)

Please give reason for your answer

**(B) Using regulatory data in Cochrane reviews generally**

Q8 B1. Do you think regulatory data should be used in Cochrane reviews?

- Yes (1)
- In some cases (2)
- No (3)
- Unsure (4)

If Unsure Is Selected, Then Skip To B3. Are there barriers to using regul...

Q9 B2. Why do you think this?

Q10 B3. Are there barriers to using regulatory data in Cochrane reviews?

- Yes (1)
- No (2)
- Unsure (3)

If Yes Is Selected, Then Skip To B4. What are these barriers and what ...If No Is Selected, Then Skip To B5. What could be done to promote and...If Unsure Is Selected, Then Skip To B4. What are these barriers and what ...

Q11 B4. What are these barriers and what would help overcome them?

Q12 B5. What could be done to promote and support greater use of regulatory data in Cochrane reviews?

**(C) Other comments**

Q13 C1. Any other comments

**(D) Your background and role**

Q14 D1. Cochrane role (please check all that apply)

- Reviewer/author (1)
- Editor (2)
- Trial Search Coordinator (3)

Q15 D2. Your background (please check all that apply)

- Statistician (1)
- Methodologist (2)
- Clinician (3)
- Acadmic researcher (4)
- Other (please specify) (5) ____________________

**(E) Next steps**

Q16 We plan to collate information about the main triggers for requesting regulatory data and then consult on how important each of these are

Q17 E1. Would you be willing to participate in a short follow-up survey about this?

- Yes (1)
- No (2)

Q18 E2. Would you like to receive a copy of our final report?

- Yes (1)
- No (2)

Q19 If yes (to either survey or report) please provide your name and email address so that we can contact/send to you

Q20 Name

Q21 Email address

Thank you for your participation

1. **Not considered regulatory data**

This survey is for Cochrane authors who have never considered using information/data from regulatory sources in a systematic review. If this criterion does not apply, please return to the invitation to participate and click on the appropriate link. Participation in this survey is voluntary

**A. Understanding of the regulatory environment**

Q1 A1. Are you familiar with the regulatory process for pharmaceuticals and biologics?

- Yes - detailed understanding (1)
- Yes - basic understanding (2)
- No (3)

If Yes - detailed understanding Is Selected, Then Skip To A2. Are you familiar with the types o...If Yes - basic understanding Is Selected, Then Skip To A2. Are you familiar with the types o...If No Is Selected, Then Skip To A3. Are you aware of the ongoing deba...

Q2 A2. Are you familiar with the types of documents produced?

- Yes very familiar (1)
- Yes have some knowledge (2)
- No (3)
- Unsure (4)

Q3 Comments

Q4 A3. Are you aware of the ongoing debate for increased access to clinical trial data?

- Yes (1)
- No (2)

Q5 Comments

Q6 A4. Do you know where to access trial regulatory data and material?

- Yes (1)
- No (2)
- Unsure (3)

If Yes Is Selected, Then Skip To A5. Please describe briefly where you...If No Is Selected, Then Skip To End of BlockIf Unsure Is Selected, Then Skip To A5. Please describe briefly where you...

Q7 A5. Please describe briefly where you think regulatory data/information can be accessed

**B. Using regulatory data in systematic reviews**

Q8 B1. Are you aware that some systematic reviews have included data from regulatory sources (in addition to or instead of data from publications)?

- Yes (1)
- No (2)
- Unsure (3)

Q9 Comments

Q10 B2. Do you think trial data from regulatory sources should be used in Cochrane reviews?

- Yes (1)
- In some cases (2)
- No (3)
- Unsure (4)

If Unsure Is Selected, Then Skip To B4. Would you consider using regula...

Q11 B3. Why do you think this?

Q12 B4. Would you consider using regulatory data in any of your Cochrane reviews?

- Yes (1)
- No (2)
- Unsure (3)

Q13 Comments

Q14 B5. Do you think there are any barriers to using regulatory data in Cochrane reviews?

- Yes (1)
- No (2)
- Unsure (3)

If No Is Selected, Then Skip To End of BlockIf Unsure Is Selected, Then Skip To End of Block

Q15 B6. What are these barriers and what would help overcome them?

**C) Other comments**

Q16 C1. Any other comments

**(D) Your background and role**

Q17 D1. Cochrane role (please tick all that apply)

- Reviewer/author (1)
- Editor (2)
- Trial Search Coordinator (3)

Q18 D2. Your background (please tick all that apply)

- Statistician (1)
- Methodologist (2)
- Clinician (3)
- Academic researcher (4)
- Other (please specify) (5) ____________________

**(E) Receiving a copy of our report**

Q19 E1. Would you like to receive a copy of our final report?

- Yes (1)
- No (2)

Q20 If yes, please provide your name and email address so that we can send this to you

Q21 Name

Q22 Email address

Q23 Final comments

Thank you for your participation
