# Supplementary material for: The use of clinical study reports to enhance the quality of systematic reviews: a survey of systematic review authors
Source: Syst Rev. 2018 Aug 8;7:117. doi: 10.1186/s13643-018-0766-x (PMC6083614; doi:10.1186/s13643-018-0766-x)
Supplement: Supplementary file 2 — Follow-up survey. (DOCX 16 kb) [file 13643_2018_766_MOESM2_ESM.docx]

**Additional file 2: Follow-up survey for those who Used/Requested/Considered regulatory data**

This survey is for people who previously used or considered using data from clinical study reports (CSRs) and/or other regulatory data in their review(s). The survey should take no more than 10 minutes to complete and participation is voluntary.

Q1 Do you think it is important to use Clinical Study Reports (CSRs) and/or regulatory data in reviews?

- Yes, always (1)
- In some cases (2)
- No, never (3)
- Unsure (4)

Condition: Yes, always is selected. Skip To: We recognize that not all Cochrane re....Condition: In some cases Is Selected. Skip To: We recognize that not all Cochrane re....Condition: No, never Is Selected. Skip To: Why do you think this? Condition: Unsure Is Selected. Skip To: We recognize that not all Cochrane re....

We recognize that not all Cochrane reviews will be able to use data from clinical study reports (CSRs) and/or other regulatory data. This survey therefore aims to identify the criteria or ‘triggers’ that may be useful in helping reviewers decide when it is most important to seek data from clinical study reports (CSRs) and/or other regulatory data.

In your opinion which of the following factors are important ‘triggers’ when considering whether or not to seek data from CSRs and/or other regulatory documents

Cost:

|  | Very important (1) | Important (2) | Less important (4) | Not important (5) | Unsure (6) |
| --- | --- | --- | --- | --- | --- |
| Monetary cost of the intervention on the healthcare budget (i.e. considering both the price of a course and the number of people in the population that are being - or will be treated)? |  |  |  |  |  |

Population/disease:

|  | Very important (1) | Important (2) | Less important (4) | Not important (5) | Unsure (6) |
| --- | --- | --- | --- | --- | --- |
| Burden of disease of the indication this product is meant to treat/prevent? |  |  |  |  |  |
| How many people are using or likely to use this product? |  |  |  |  |  |

Product/Intervention:

|  | Very important (1) | Important (2) | Less important (4) | Not important (5) | Unsure (6) |
| --- | --- | --- | --- | --- | --- |
| Product new to the market? |  |  |  |  |  |
| Product from a new drug class or has a new mechanism of action |  |  |  |  |  |
| Has important interactions with other drugs (e.g. drug-drug interactions) |  |  |  |  |  |
| High proportion of RCTs evaluating this product are industry funded |  |  |  |  |  |
| Prominent claims of safety and/or efficacy advantage of this product over currently available treatments |  |  |  |  |  |
| High degree of media attention surrounding this product |  |  |  |  |  |

Reporting Bias:

|  | Very important (1) | Important (2) | Less important (4) | Not important (5) | Unsure (6) |
| --- | --- | --- | --- | --- | --- |
| High proportion of trials of this product are unpublished |  |  |  |  |  |
| Post-marketing surveillance has identified safety concerns? |  |  |  |  |  |
| Important or standard outcome measures (also known as 'endpoints') have not been published |  |  |  |  |  |
| Concerns regarding a lack of published data on potential harms of the product |  |  |  |  |  |
| Marketing authorization based on surrogate outcomes (rather than clinical outcomes) |  |  |  |  |  |
| Known errors or concerns about trial publications of this product |  |  |  |  |  |
| When protocol(s) are publicly available |  |  |  |  |  |
| When statistical analysis plan(s) publicly available? |  |  |  |  |  |
| Important discrepancies between the journal publication and the trial registry entry? |  |  |  |  |  |
|  |  |  |  |  |  |

Q2 What other questions/criteria do you consider important to help decide whether or not to seek data from clinical study reports (CSRs) and/or other regulatory data?

Q3 Any further comments you would like to make?

Condition: Comments Is Displayed. Skip To: End of Block.

Q4 Why do you think this?

We plan to combine this information with data collected from our earlier survey into a single report. We will then consult on findings and proposed recommendations before going on to develop proposed guidance about when it might be most important to seek data from clinical study reports and/or other regulatory data in Cochrane reviews.

Q5 Name

Q6 Email address
