# Supplementary material for: The use of clinical study reports to enhance the quality of systematic reviews: a survey of systematic review authors
Source: Syst Rev. 2018 Aug 8;7:117. doi: 10.1186/s13643-018-0766-x (PMC6083614; doi:10.1186/s13643-018-0766-x)
Supplement: Supplementary file 3 — Table S1. Characteristics of respondents and their experiences with regulatory data. Table S2.Characteristics of respondents who have never considered using regulatory data. Table S3. Possible sources for respondents who had not considered regulatory data. (DOCX 17 kb) [file 13643_2018_766_MOESM3_ESM.docx]

**Additional file 3: Appendix tables**

**Additional file 3 – Table S1: Characteristics of respondents and their experiences with regulatory data**

|  | **Requested regulatory data** | **Considered regulatory data** | **Not considered regulatory data** |
| --- | --- | --- | --- |
| **Question** | **Total no. of responses: n (% of total responses)** | | |
| Should regulatory data be used in Cochrane reviews? | n=20 | n=7 | n=133 |
| Yes | 15 (75) | 3 (43) | 43 (32) |
| In some cases | 5 (25) | 3 (43) | 66 (50) |
| No | 0 (0) | 0 (0) | 17 (13)^β^ |
| Unsure | 0 (0) | 1 (14) | 7 (5) |
| Rationale for using regulatory data? | n=20 | n=7 | N/A |
| Under reporting of harms | 3 | 2 | N/A |
| ORB | 11 | 3 | N/A |
| Publication bias | 5 | 0 | N/A |
| Missing data | 2 | 1 | N/A |
| Other | 2* | 2** | N/A |

N/A: question was not asked in the survey as it was none applicable; ORB: outcome reporting bias

*(n=1) was a request from a reviewer and, (n=1) for detailed medical information from manufacturer about the product

**(n=1) over uncertainty in quality assessment domains and (n=1) basis on national Australian guidelines

^β^ Reasons why regulatory data should not be considered: (n=9) interventions non-pharmacological, (n=5) lack of guidance on how to include the data and (n=3) too time consuming

**Additional file 3 – Table S2: Characteristics of respondents who have never considered using regulatory data**

| **Question** | **Not considered regulatory data** |
| --- | --- |
| Familiarity with the regulatory process for pharmaceutical and biologics? | n=133 |
| Yes - detailed understanding | 8 (6) |
| Yes - basic understanding | 83 (62) |
| No | 42 (32)^γ^ |
| Awareness of debate for improved access to clinical trial data? | n=133 |
| Yes | 113 (85)*** |
| No | 20 (15) |

^γ^ Reason not familiar: (n=2) Respondents conduct non-pharmacological reviews that do not require familiarity with regulatory data

***(n=2) respondents mentioned the AllTrials initiative, (n=2) mentioned the Tamiflu review by Jefferson et al. 2009, (n=1) respondent was involved in the EMAs policy 70 act for access to clinical trial data in 2014 and (n=1) Ben Goldacre’s Bad pharma.

**Additional file 3 – Table S3: Possible sources for respondents who had not considered regulatory data**

| **Question** | **Not considered regulatory data** |
| --- | --- |
| Do you know where to access trial regulatory data? | n=133 |
| Yes | 16 (12) |
| No | 77 (58) |
| Unsure | 40 (30) |
| Where do you think regulatory data/information are available? | n=47 |
| FDA | 19 |
| EMA | 18 |
| Other regulators | 4^β^ |
| Trial registries | 10^€^ |
| Pharmaceutical company | 10 |
| NICE | 3 |
| MHRA | 5 |
| Clinical Study Data Requests website | 1 |
| Ethics committee | 3 |
| Research council (MRC) | 1 |
| Governmental body | 2 |
| Are authors familiar with the type’s documents produced? | n=91 |
| Yes very familiar | 3 (3)^π^ |
| Yes have some knowledge | 51 (56)^α^ |
| No | 26 (29) ^γ^ |
| Unsure | 11 (12) |

N/A: question was not applicable to the questionnaire; EMA: European medicines agency; FDA: food and drug administration; NICE: national institute for health and care excellence; MHRA: the Medicines and Healthcare products Regulatory Agency

^β^ The Health Products Regulatory Authority (HPRA) of Ireland, Pharmaceuticals and medical devices agency (PMDA) of Japan, Therapeutic Good Administration (TGA) Department of Health Australia (listed twice)

^γ^ Reason no familiarity: (n=3) respondents conduct non-pharmacological reviews that do not require familiarity with document types for regulatory data

^€^ (n=9) ClinicalTrials.gov website and (n=2) international standard randomised controlled trial number (ISRCTN) registry.

^π^ One respondent had previous experience using open data sources from EMA

^α^ One respondent was a member of a regional ethical committee, and therefore is aware of the type of documents produced at that level, another respondent had worked for the Italian drug agency and has some expertise on the content of dossiers submitted for regulatory purposes, and one author has previous experience writing periodic safety update reports.
